# Association between Chinese visceral adiposity index and risk of new-onset hypertension in middle-aged and older adults with prediabetes: evidence from a large national cohort study

**DOI:** 10.3389/fpubh.2025.1509898

**Published:** 2025-02-12

**Authors:** Lanlan Li, Linqiang Xi, Qianhui Wang

**Affiliations:** ^1^Department of Nephrology, Hospital of Xinjiang Production and Construction Corps, Urumqi, Xinjiang, China; ^2^Department of Nephrology, Second Affiliated Hospital, Medical School of Shihezi University, Urumqi, Xinjiang, China; ^3^Department of Cardiac Pacing and Electrophysiology, The First Affiliated Hospital of Xinjiang Medical University, Urumqi, China; ^4^Department of Cardiology, The Sixth Affiliated Hospital of Jinan University (Dongguan Eastern Central Hospital), Dong Guan, Guangdong, China

**Keywords:** prediabetes, hypertension, obesity, Chinese visceral adiposity index, biomarker

## Abstract

**Purpose:**

Chinese Visceral Adiposity Index (CVAI) has been found significantly associated with hypertension in general and type-2 diabetes adults. However, the predictive value of CVAI for the incidence of hypertension in adults with prediabetes is unclear. This study aimed to assess the predictive utility of the CVAI for the new onset of hypertension in middle-aged and older adult Chinese individuals with prediabetes.

**Methods:**

A prospective cohort study was conducted involving participants aged 45 years and above with prediabetes from the 2011–2012 cohort of the China Health and Retirement Longitudinal Study (CHARLS). Logistic regression models were utilized to investigate the association between CVAI levels and the risk of new-onset hypertension.

**Results:**

The study included 2,186 participants, among whom 444 (20.31%) developed hypertension. Significantly higher incidence rates of hypertension were observed in individuals belonging to the highest quartile group (Q4) compared to those in the lowest quartile group (Q1) of CVAI (29.41% vs. 14.69%, *p* < 0.001). Multivariate logistic regression analysis indicated that participants in Q4 had a 1.91-fold greater risk of hypertension development compared to those in Q1 (odds ratio (OR): 1.91, 95% confidence interval (CI): 1.49–2.45, *p* < 0.001). The area under the receiver operating characteristic (ROC) curve (AUC) demonstrated that CVAI exhibited superior performance in discriminating individuals at heightened risk of hypertension compared to other obesity-related indices (*p* < 0.001). A subgroup analysis revealed that age may modulate the relationship between CVAI and new-onset hypertension, with a more pronounced interaction observed among participants below 60 years of age (*P* for interaction: 0.026).

**Conclusion:**

Elevated CVAI levels were significantly associated with an increased risk of developing hypertension. CVAI proves to be a reliable and effective tool for risk stratification in middle-aged and older adult Chinese individuals with prediabetes, underscoring its substantial implications for primary prevention of hypertension and public health strategies.

## Introduction

Hypertension, characterized by consistently elevated blood pressure (BP) levels, stands as a primary risk factor for cardiovascular disease (CVD) and mortality in middle-aged and older adult individuals (1, 2). A nationwide survey conducted in China in 2012 revealed that approximately 23.2% of adults were afflicted with hypertension (3), with estimates suggesting this prevalence could reach up to half among individuals aged 35–75 years (4). The aging demographic has witnessed a notable surge in cases of hypertension, with projections indicating that by 2025, roughly one-third of the global population will grapple with hypertension (5). Despite its pervasive presence in China, the rates of awareness, drug therapy, and effective control of hypertension remain suboptimal, necessitating urgent attention toward early and precise identification of risk factors to preemptively thwart its onset and alleviate the associated burden.

Recent studies have emphasized the importance of prediabetes, characterized by consistently elevated blood glucose levels below the diabetic threshold, in increasing the risk of developing type-2 diabetes and CVD (6, 7). Specifically, a cohort study conducted by Yue et al. (8) in Northeast China, which recruited adults aged 40 and above, demonstrated that individuals in this age group with both hypertension and prediabetes faced a significantly higher risk of CVD mortality compared to those with prediabetes alone. Therefore, identifying individuals with prediabetes who are at a heightened risk of progressing to hypertension is of paramount importance.

Obesity, typified by excessive fat accumulation, has emerged as a strong correlation of hypertension development (9, 10). Amidst China’s rapid economic growth in recent decades, the prevalence of overweight and obesity has surged among adults and adolescents, posing substantial challenges to public health systems (11). Studies have highlighted the association between elevated BP and excessive visceral adipose tissues (VAT), particularly over subcutaneous or total fat (12, 13). However, accurately gauging VAT in large-scale studies poses challenges due to factors like radiation exposure risks, time constraints, and the prohibitive costs linked with gold standard methods such as computed tomography (CT) or magnetic resonance imaging (MRI). Conventional anthropometric measures like body mass index (BMI) and waist circumference (WC) fall short in precisely assessing abdominal fat distribution.

Originally devised based on Caucasian populations, the visceral adiposity index (VAI) has emerged as a dependable proxy for evaluating VAT dysfunction and is significantly tied to an escalated risk of hypertension in Western populations (14, 15). Nevertheless, the utility of VAI in appraising VAT has been found wanting in Asian subjects owing to differing body fat distribution characteristics. The Chinese visceral adiposity index (CVAI), a novel all-encompassing surrogate VAT index tailored for Chinese adults, integrates demographic factors (age), anthropometric metrics (BMI and WC), and lipid profiles (serum triglyceride (TG) and high-density lipoprotein (HDL)) (16). CVAI has exhibited strong alignment with CT validation outcomes and has been linked to a markedly increased risk of CVD (17), hypertension (18), and diabetes (19).

The question remains as to whether CVAI exhibits a positive association with the risk of new-onset hypertension in middle-aged and older adult populations with prediabetes. This study seeks to explore the prospective predictive value of CVAI for new-onset hypertension and assess its efficacy relative to other obesity and insulin resistance-related indices (including triglyceride-glucose index (TyG), TyG-BMI, WC to height ratio (WHtR), BMI and WC) using data from the China Health and Retirement Longitudinal Study (CHARLS).

## Methods

### Study design and participants

This is a prospective cohort study that utilizes data sourced from CHARLS, a nationally representative longitudinal survey overseen by the National School of Development at Peking University and funded by the US National Institute on Aging (NIA). The study targeted community residents aged 45 and above in China. Data collection involved computer-assisted personal interviews (CAPI) and structured questionnaires to capture a comprehensive range of health and retirement-related factors from the participants. The study was initiated in 2011, with subsequent follow-ups conducted biennially. To date, data from three waves (Wave 2: 2013, Wave 3: 2015, and Wave 4: 2018) have been made publicly accessible on the website http://charls.pku.edu.cn/. However, Wave 4 data was not utilized in this study due to lacking information on anti-hypertensive medication usage and BP tests.

**Figure 1:**
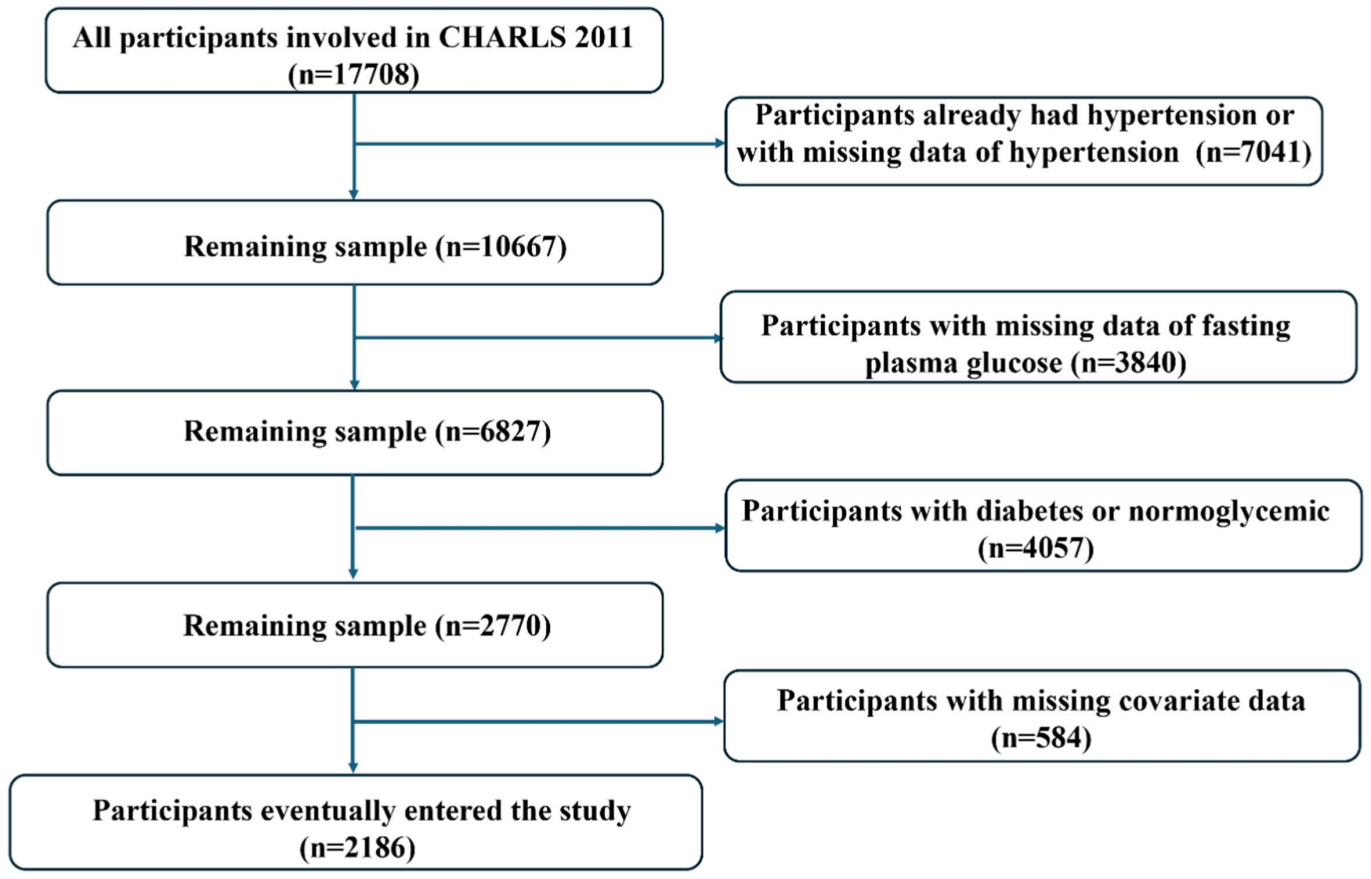
Flow chart of participant selection.

Participants were excluded from the study if they met any of the following criteria: (1) with hypertension at baseline; (2) inadequate follow-up data on hypertension, including physician diagnosis, use of anti-hypertensive medications, and BP tests; (3) mortality during the follow-up period; (4) missing baseline covariate data; or (5) existing diabetes or normoglycemia at baseline (Figure 1). The survey received approval from the Ethics Review Board of Peking University, and all participants provided written informed consent.

### Definition

Hypertension was defined based on the presence of any of the following criteria: self-reported physician diagnosis of hypertension, recent use of antihypertensive medication, or systolic BP (SBP) ≥140 mm Hg and/or diastolic BP (DBP) ≥90 mm Hg (1).

Prediabetes was characterized by fasting plasma glucose (FPG) levels between 100 and 125 mg/dL or glycosylated hemoglobin A1c (HbA1c) levels between 5.7 and 6.4%. Diabetes was identified by FPG levels ≥125 mg/dL, HbA1c levels ≥6.5%, self-reported history of diabetes, or usage of antidiabetic medications (20).

### Measurements

#### CVAI

−267.93 + 0.68 × age (years) + 0.03 × BMI (kg/m^2^) + 4.00 × WC (cm) + 22.00 × log10 (TG) (mmol/L) – 16.32 × HDL-C (mmol/L) (Male).

−187.32 + 1.71 × age (years) + 4.23 × BMI (kg/m^2^) + 1.12 × WC (cm) + 39.76 × log10 (TG) (mmol/L) – 11.66 × HDL-C (mmol/L) (Female).

#### VAI

WC (cm) / (39.68 + 1.88 × BMI) × TG (mmol/L)/1.03 × 1.31/HDL-C (mmol/L) (Male).

WC (cm) / (36.58 + 1.889 × BMI) × TG (mmol/L)/0.81 × 1.52/HDL-C (mmol/L) (Male).

TyG: Ln [fasting triglycerides (mg/dl) × fasting glucose (mg/dL)/2].

TyG-BMI: Ln [fasting triglycerides (mg/dl) × fasting glucose (mg/dL)/2] × BMI.

WHtR: WC (cm)/ height (cm).

### Covariates data collection

The study gathered sociodemographic and lifestyle data, including age, gender, education levels, marital status, smoking status, and alcohol consumption. Laboratory tests encompassed total cholesterol (TC), low-density lipoprotein (LDL), TG, HDL, uric acid (UA), blood creatine, and C-reactive protein (CRP). Estimated glomerular filtration rate (eGFR) was computed using a specific formula: 186 × (creatine)^−1.154^ × (age)^−0.203^ × 0.742 (if female). Additionally, chronic diseases such as CVD (self-reported heart disease or stroke) and hyperlipidemia (self-reported or taking lipid-lowering agents or TC ≥ 240 mg/dL or LDL ≥ 160 mg/dL or TG ≥ 200 mg/dL or HDL < 40 mg/dL) were recorded.

### Statistical analysis

In this study, individuals were categorized into four subgroups based on quartiles of the CVAI: Q1 (CVAI≤61.46), Q2 (61.46 < CVAI≤85.81), Q3 (85.81 < CVAI≤110.89), and Q4 (CVAI>110.89). Continuous variables were represented as mean ± standard deviation (SD) or median (interquartile range IQR), dependent on the data distribution, and tested using t-tests or Mann–Whitney U tests between the two groups as appropriate. One-way ANOVA or Kruskal–Wallis tests were used among the four groups. Categorical data were presented as percentages (%) and analyzed using Chi-square tests.

Three logistic regression models were developed to assess the odds ratio (OR) and 95% confidence interval (CI) for new-onset hypertension in relation to CVAI, examining both its continuous impact (per IQR increment) and its categorical effect across quartiles. Model I was unadjusted; Model II was adjusted for gender, education levels, marital status, residential location, smoking status, and alcohol consumption; Model III included additional adjustments for CVD, hyperlipidemia, CRP, LDL, TC, and e-GFR.

To explore the nonlinear association and dose–response relationship between CVAI and new-onset hypertension, a restricted cubic spline (RCS) analysis was conducted that incorporated 4 knots at 5th, 35th and 65th and 95th percentiles of CVAI. Subgroup analyses, stratifying by age, gender, education levels, smoking status, and CVD, were performed to evaluate the robustness of the association across different populations. Interaction analyses were also carried out to identify potential moderating effects among these factors.

Receiver operating characteristic (ROC) curves were utilized to assess the diagnostic utility of CVAI for predicting new-onset hypertension among individuals with prediabetes. Furthermore, a *Z*-test was employed to compare the diagnostic accuracy of CVAI against other indices related to obesity and insulin resistance in predicting new-onset hypertension.

Statistical analyses were conducted using STATA (version 14.0) and R studio (version 4.0.3), with a significance threshold set at *p* < 0.05 to determine statistical significance. All figures in this study were drawn using GraphPad Prism 8.0.

## Results

### Baseline characteristics of participants

**Figure 2:**
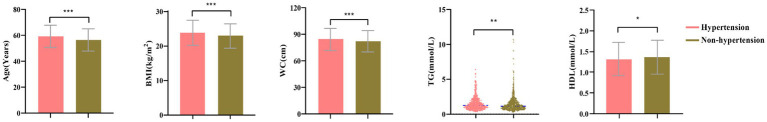
Comparison of the data from the CVAI formulas between those with hypertension and without hypertension. *p < 0.05, **p < 0.01, ***p < 0.001.

A total of 2,186 participants were ultimately included in this study, with hypertension developing in 444 individuals (20.31%). The baseline characteristics of the participants are detailed in Table 1 and Figure 2. Among those with prediabetes, 1,016 (46.48%) were males, with an average age of 57.10 ± 8.59 years. Individuals who developed hypertension tended to be older, obese, less educated, and exhibited higher levels of SBP, DBP, TG and UA and lower level of HDL compared to those who did not develop hypertension.

**Table 1:** Baseline characteristics of participants.

Variables	Total (*n* = 2,186)	Non-hypertension (*n* = 1742)	Hypertension (*n* = 444)	*p*-value
Male (%)	46.48	45.75	49.32	0.178
Marital status (%)				0.205
Married	86.09	86.57	84.23	
Others	13.91	13.43	15.77	
Education, (%)				0.042
Less than middle school	68.98	67.74	73.87	
Middle school	21.18	21.93	18.24	
High school or above	9.84	10.33	7.88	
Residence, (%)				0.912
Village	31.98	67.97	68.24	
City	68.02	32.03	31.76	
Smoking, (%)				0.341
Non-smoker	61.62	61.77	61.04	
Ex-smoker	8.23	7.81	9.91	
Current smoker	30.15	30.42	29.05	
Alcohol consumption (%)	38.15	37.37	41.22	0.076
SBP (mmHg)	118.07 ± 11.53	116.41 ± 11.27	124.56 ± 10.19	<0.001
DBP (mmHg)	70.52 ± 8.85	69.64 ± 8.81	73.99 ± 8.16	<0.001
CVD (%)	9.56	9.07	11.45	0.075
e-GFR (ml/min/1.73 m^2^)	100.44 ± 23.53	100.87 ± 23.54	98.74 ± 23.42	0.088
TC (mg/dL)	195.00 ± 38.24	194.46 ± 38.20	197.12 ± 38.37	0.192
LDL-C (mg/dL)	118.05 ± 35.25	117.97 ± 34.96	118.37 ± 36.41	0.834
CRP (mg/L)	0.90 (0.52, 1.88)	0.90 (0.51, 1.85)	0.93 (0.56, 2.02)	0.273
UA (mg/dL)	4.35 ± 1.97	4.32 ± 1.18	4.46 ± 1.25	<0.034
CVAI	86.04 ± 41.35	83.35 ± 40.78	96.60 ± 41.94	<0.001
VAI	1.39 (0.86, 2.53)	1.37 (0.86, 2.40)	1.63 (0.91, 2.94)	0.005
WHtR	0.52 ± 0.08	0.52 ± 0.08	0.54 ± 0.08	<0.001
TyG index	8.67 ± 0.55	8.65 ± 0.54	8.76 ± 0.58	<0.001
TyG-BMI	200.61 ± 36.51	198.47 ± 35.73	209.02 ± 38.34	<0.001

SBP, systolic blood pressure; DBP, diastolic blood pressure; e-GFR, estimated glomerular filtration rate; TC, total cholesterol; TG, triglyceride; HDL-C, High-density lipoprotein cholesterol; LDL-C, Low-density lipoprotein cholesterol; CRP, C-reactive protein; UA, uric acid; CVAI, Chinese visceral adiposity index; VAI, visceral adiposity index; BMI, Body mass index; WC, Waist circumference; WHtR, waist to height ratio; TyG, triglyceride-glucose index; TyG-BMI, triglyceride-glucose-body mass index.

### Baseline characteristics of participants based on the quartiles of CVAI

Table 2 presents the baseline characteristics of the participants categorized into quartiles based on CVAI. Individuals with higher CVAI levels were generally older, predominantly female, residing in urban areas, and had a history of cardiovascular disease. They had a lower prevalence of smoking, but higher alcohol consumption. Furthermore, they exhibited elevated levels of SBP, DBP, LDL-C, CRP, UA, and a lower e-GFR. Notably, there was a significant increase in the incidence of hypertension as CVAI quartiles rose (Q1: 14.69% vs. Q2: 14.81% vs. Q3: 23.89% vs. Q4: 29.41% *p* < 0.001).

**Table 2:** Baseline characteristics of participants across quartiles of CVAI.

Variables	Q1	Q2	Q3	Q4	*p*
Age (Year)	55.15 ± 8.53	56.61 ± 8.24	57.80 ± 8.80	59.11 ± 8.35	<0.001
Male (%)	56.86	43.56	38.52	47.26	<0.001
Marital Status (%)					0.378
Married	84.28	86.30	85.92	88.03	
Others	15.72	13.70	14.08	11.97	
Education (%)					0.183
Less than middle school	68.01	71.12	67.96	68.56	
Middle school	23.22	21.12	20.37	19.88	
High school or above	8.78	7.76	11.67	11.56	
Residence (%)					<0.001
Village	73.67	72.44	66.30	58.22	
City	26.33	27.56	33.70	41.78	
Smoking status (%)					<0.001
Non-smoker	53.02	62.54	68.33	62.68	
Ex-smoker	6.58	4.62	9.26	13.39	
Current smoker	40.40	32.84	22.41	23.94	
Alcohol consumption (%)	36.96	42.60	32.22	41.18	0.002
SBP (mmHg)	115.41 ± 12.18	117.05 ± 11.39	118.71 ± 10.87	121.59 ± 10.72	<0.001
DBP (mmHg)	68.58 ± 9.33	69.79 ± 8.54	71.08 ± 8.43	72.97 ± 8.52	<0.001
Hypertension (%)	14.69	14.81	23.89	29.41	<0.001
CVD (%)	7.59	6.95	10.19	14.20	<0.001
e-GFR (ml/min/1.73 m^2^)	103.70 ± 22.71	102.58 ± 24.20	97.44 ± 22.76	97.47 ± 23.70	<0.001
TC (mg/dL)	188.64 ± 36.96	194.22 ± 38.90	196.22 ± 37.34	201.68 ± 38.70	<0.001
LDL-C (mg/dL)	111.95 ± 32.39	118.34 ± 34.18	121.38 ± 35.57	120.84 ± 38.32	<0.001
CRP (mg/L)	0.65 (0.40, 1.41)	0.80 (0.49, 1.62)	1.05 (0.60, 2.14)	1.25 (0.71, 2.34)	<0.001
UA (mg/dL)	4.19 ± 1.15	4.17 ± 1.12	4.34 ± 1.21	4.77 ± 1.22	<0.001

CVAI, Chinese visceral adiposity index; SBP, systolic blood pressure; DBP, diastolic blood pressure; e-GFR, estimated glomerular filtration rate; TC, total cholesterol; LDL-C, Low-density lipoprotein cholesterol; CRP, C-reactive protein; UA, uric acid.

### Association between CVAI and new-onset hypertension

**Figure 3:**
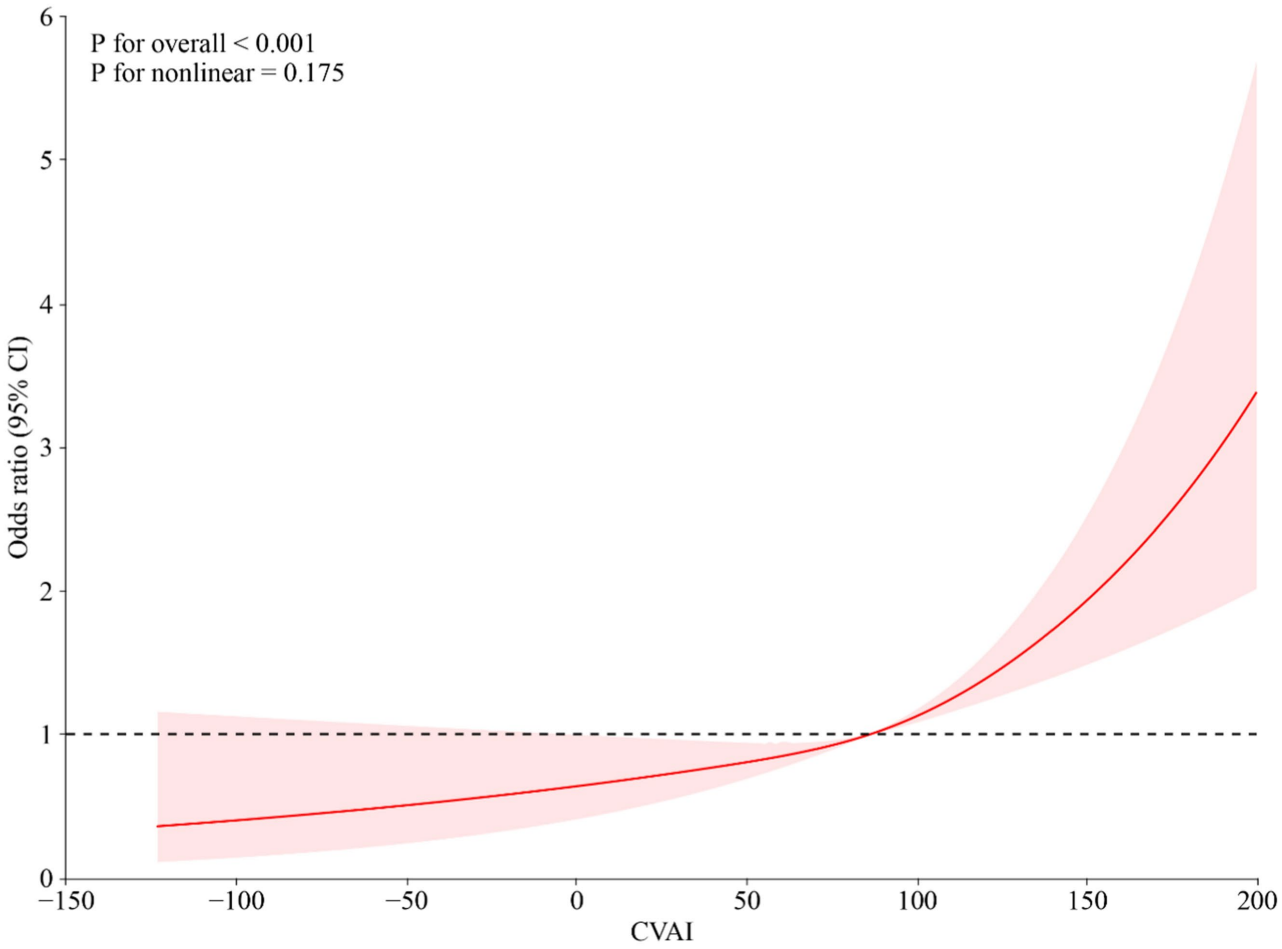
Restricted cubic spline for the incidence of hypertension by CVAI after adjusting for confounding factors.

Table 3 illustrated that per 1-IQR increase in CVAI was significantly linked to a 26% higher risk of developing hypertension among middle-aged and older adults with prediabetes (OR: 1.26, 95% CI: 1.16–1.36). Furthermore, participants in the third (Q3) and fourth (Q4) quartiles of CVAI faced a 37% (OR: 1.37, 95% CI: 1.07–1.75) and a 91% (OR: 1.91, 95% CI: 1.49–2.45) increased the risk of developing hypertension, respectively, compared to those in the first quartile (Q1). Multivariable adjusted restricted cubic spline analyses revealed a significant linear dose–response relationship between CVAI and new-onset hypertension (*P* for overall: <0.001, *P*_non-linear_:0.175) (Figure 3).

**Table 3:** Association between CVAI and new-onset hypertension.

	Model I	Model II	Model III
CVAI per IQR increment	1.39 (1.30, 1.50) <0.001	1.41 (1.30, 1.51) <0.001	1.26 (1.16, 1.36) <0.001
Q1	Reference	Reference	Reference
Q2	1.13 (0.88, 1.45) 0.357	1.15 (0.89, 1.47) 0.288	1.03 (0.79, 1.34) 0.838
Q3	1.58 (1.25, 1.99) <0.001	1.64 (1.29, 2.07) <0.001	1.37 (1.07, 1.75) 0.011
Q4	2.60 (2.07, 3.27) <0.001	2.67 (2.12, 3.37) <0.001	1.91 (1.49, 2.45) <0.001
*P* for trend	<0.001	<0.001	<0.001

Data were presented as odds ratio (95% confidence interval) and *p* value.

Model I: crude model; Model II was adjusted for gender, marital status, smoking status, education levels and location; Model III was further adjusted for low-density lipoprotein cholesterol, total cholesterol, C-reactive protein, uric acid, systolic blood pressure, diastolic blood pressure, and CVD.

### Subgroup analysis

**Figure 4:**
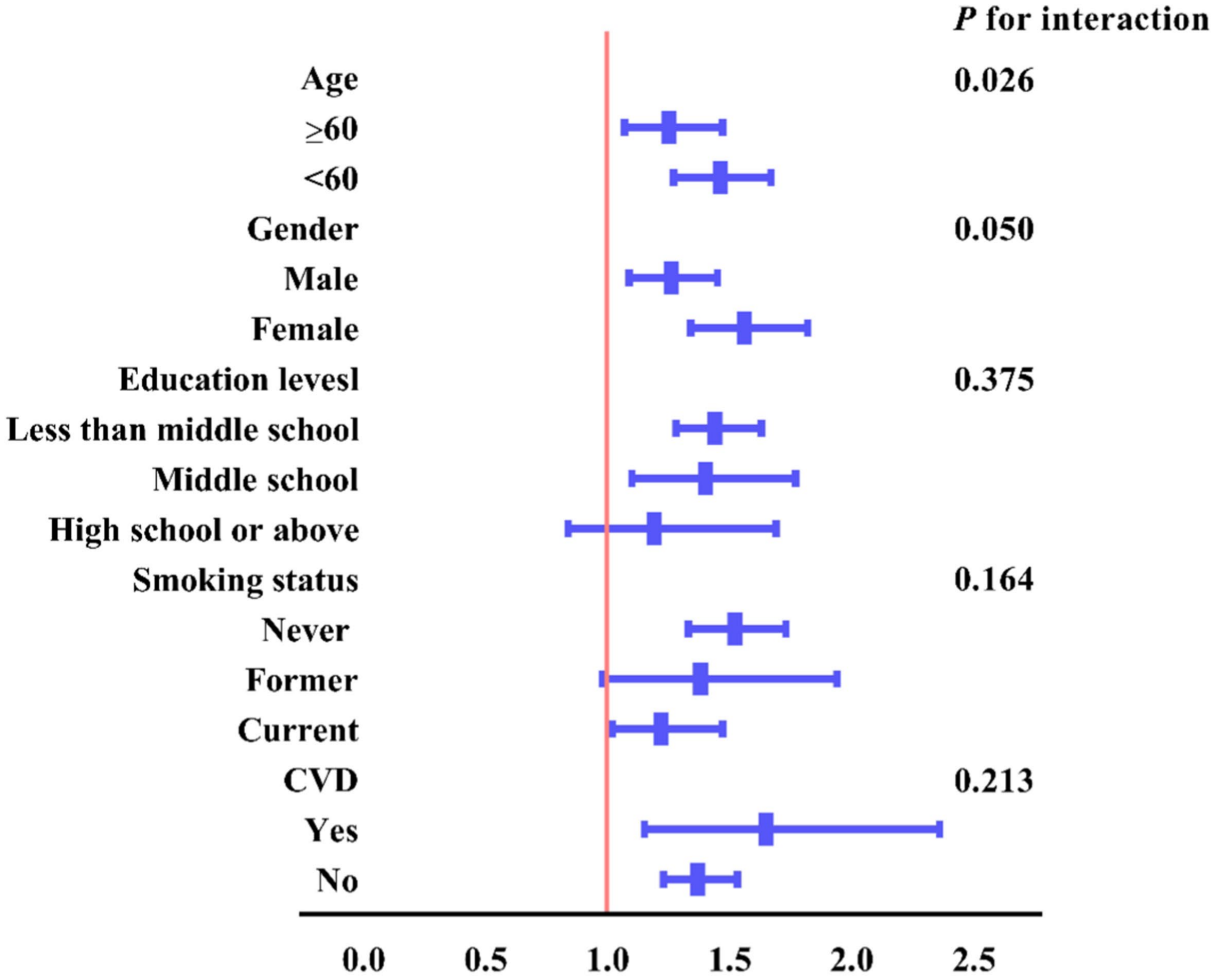
Subgroup and interaction analyses between the CVAI (per 1.0-SD increment) and hypertension across various subgroups.

Subgroup analyses were conducted to explore the association between CVAI and new-onset hypertension in various populations. As illustrated in Figure 4, age significantly modified the relationship between CVAI and new-onset hypertension among Chinese middle-aged and older adults with prediabetes (interaction *p* = 0.026). This indicated that CVAI was associated with a higher risk of new-onset hypertension among younger participants (≥60 years vs. <60 years: OR: 1.25, 95% CI: 1.07–1.47 vs. OR: 1.46, 95% CI: 1.27–1.67).

### Predictive value of CVAI and other obesity and insulin-resistance indices for the new-onset hypertension

**Figure 5:**
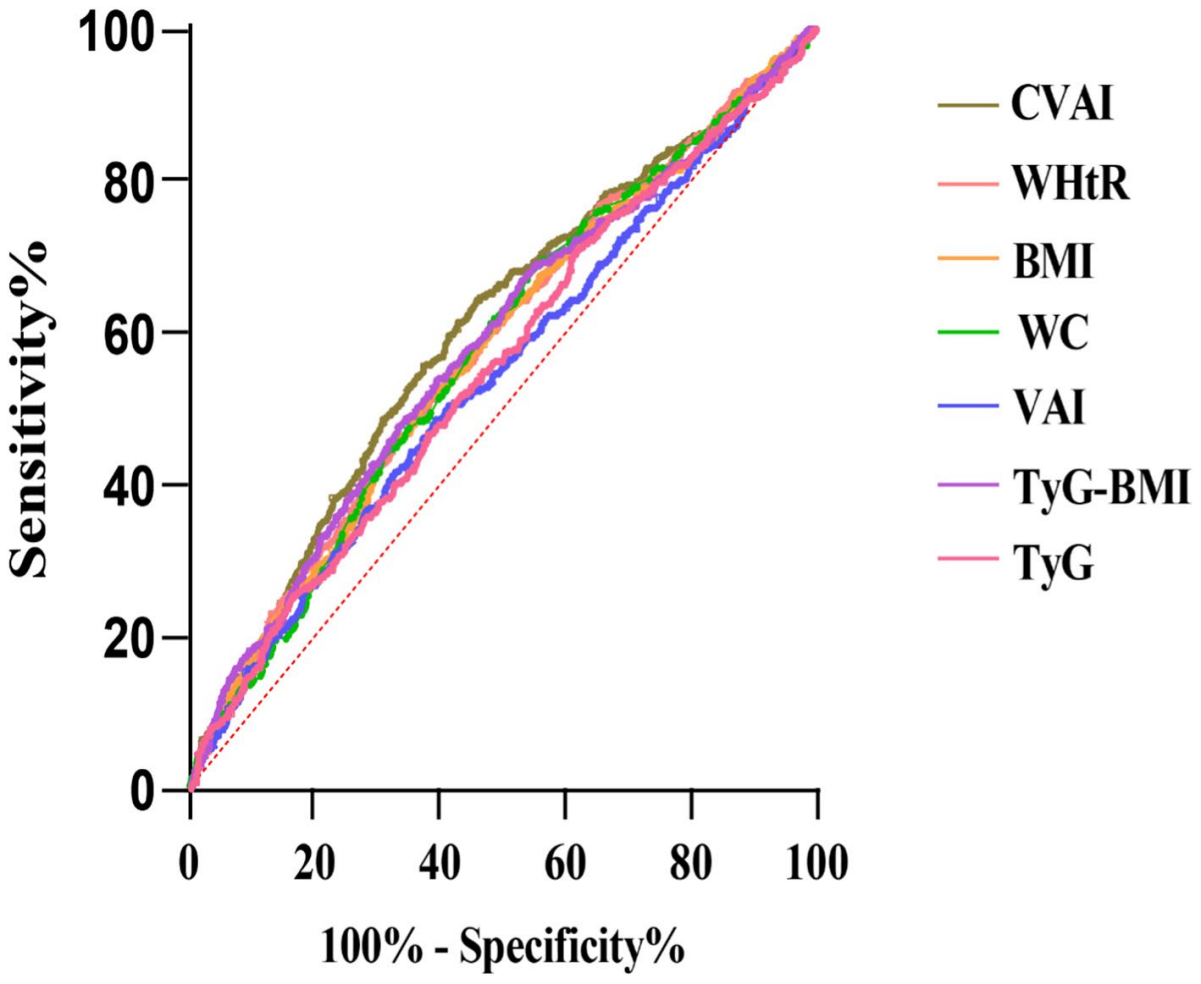
Receiver operating characteristic curves of obesity and lipids related indices in predicting hypertension.

The study also assessed the predictive capacity of CVAI and other indices for new-onset hypertension by constructing ROC curves. As shown in Figure 5, CVAI demonstrated superior predictive value for new-onset hypertension compared to other indices (area under curve (AUC) *p* < 0.05).

## Discussion

To the best of our knowledge, this study is the first to investigate the predictive value of CVAI for the development of hypertension in middle-aged and older Chinese individuals with prediabetes. The main findings of this study are as follows: (1) Participants with higher CVAI levels had a significantly increased risk of developing hypertension, even after adjusting for potential confounding factors. (2) There was a linear dose–response relationship between CVAI and the incidence of hypertension. (3) CVAI demonstrated superior predictive ability for new-onset hypertension compared to other indices related to obesity and insulin resistance.

Currently, there exists inconsistency in the diagnostic criteria for prediabetes. The World Health Organization (WHO) defines prediabetes as FPG levels between 6.1 mmol/L and 6.9 mmol/L (110-125 mg/dL) (21). On the other hand, the American Diabetes Association (ADA) recommends a lower cutoff value for FPG (5.6–6.9 mmol/L or 100–125 mg/dL) or hemoglobin A1c (HbA1c) levels of 5.7–6.4% to diagnose prediabetes (20). In this study, we adopted the ADA criteria for prediabetes diagnosis, as previous studies have shown that individuals diagnosed with prediabetes according to ADA criteria have a significantly increased risk of CVD, cardiovascular events, and mortality.

Prediabetes represents a significant risk factor for the development of diabetes, with approximately 5–10% of individuals with prediabetes progressing to diabetes within 1 year, which is significantly higher than the general population’s annual progression rate of around 3.5% (22). In our study, we observed a higher prevalence of prediabetes in rural populations compared to urban individuals (68.02% vs. 31.98%, *p* < 0.001), which is consistent with a previous cohort study conducted in Northeast China (8).

With the rapid economic and technological development in recent years, obesity, particularly abdominal obesity, has become a significant public health burden in China. An epidemiological study reported an overall obesity rate of 15.76% (BMI ≥28.0 kg/m^2^ for both women and men) and an abdominal obesity rate of 38.65% (WC ≥ 85 cm for women or WC ≥90 cm for men) among middle-aged and older Chinese individuals (23). Visceral obesity, characterized by excessive VAT accumulation, has been found to be strongly associated with an increased risk of cardiovascular disease compared to overall obesity. While WC indeed provides some insights into visceral obesity, its ability to distinguish between subcutaneous and visceral fat levels is limited (24). VAI, initially developed by Amato et al. (14) in Western populations, has been found to be significantly associated with an increased risk of metabolic syndrome (MetS) components, cardiovascular and cerebrovascular events, making it a valuable indicator for assessing visceral obesity function and insulin sensitivity. However, it’s important to note that there are notable variations in body fat percentage and distribution across different racial groups. Asians, in particular, exhibit higher body fat percentage at lower BMI compared to Caucasians and are more prone to accumulating visceral fat (25). Therefore, CVAI, a more comprehensive index, integrating age, HDL, LDL, BMI, and WC, was developed specifically for Chinese populations. In a prospective cohort study involving rural Chinese adults, elevated CVAI levels at baseline and over time were found to be significantly associated with an increased risk of developing hypertension in both males and females. Furthermore, CVAI demonstrated superior predictive capabilities for hypertension compared to other indices of visceral obesity in both genders (26). Cheng et.al (27) also found that CVAI had the best discriminative value for hypertension in patients with type-2 diabetes compared to other abdominal obesity-related indices. Consistent with these findings, our study also revealed a significant association between elevated CVAI levels and an increased risk of developing hypertension in middle-aged and older Chinese individuals with prediabetes. Additionally, CVAI outperformed other obesity and lipid-related indices in identifying individuals at higher risk for developing hypertension.

The detailed mechanisms linking visceral obesity to the development of hypertension in individuals with prediabetes are still not fully understood and warrant further investigation. However, potential explanations include: (1) Upregulated activity of the renin-angiotensin aldosterone system (RAAS) induced by adipose tissue, which may significantly contribute to the development of hypertension in adults with prediabetes. The local RAAS plays a crucial role in adipocyte differentiation and triglyceride modulation (28). Adipocytes secrete Angiotensin II type 2 receptors, which promote the proliferation of mature, insulin-sensitive adipocytes and the differentiation of preadipocytes (29). A prediabetes animal model induced by a high-fat, high-carbohydrate diet showed significantly increased expression of renin, angiotensinogen (AGT), and angiotensin II type 1 receptor (AT1R) in adipose tissue, and importantly, all components of RAAS were significantly increased in the heart in the prediabetes group (30). (2) Insulin resistance, a common pathophysiological process in the development of prediabetes and diabetes, significantly contributes to the pathogenesis of hypertension through various mechanisms in adults with prediabetes, including endothelial dysfunction, sodium retention, heightened sympathetic activity, and vascular hypertrophy (31, 32). An animal study using aged mice found that decreased accumulation of visceral fat was associated with alleviated insulin resistance (33). In our study, we also observed a significant positive correlation between CVAI and TyG-BMI, a well-established surrogate marker of insulin resistance (Pearson analysis: *r* = 0.70, *p* < 0.001).

## Limitations

Several limitations should be acknowledged in this study. Firstly, although we observed a significant association between CVAI and the onset of hypertension, the nature of observational study precludes us from establishing a causal relationship. Secondly, the study participants were exclusively Chinese, and therefore, the generalizability of the findings to other ethnic populations, particularly Western adults, warrants further investigation. Thirdly, in this study, we only included adults aged 45 years or older, which restricts the generalizability of our findings to younger populations. Future research should consider including a broader age range of Chinese individuals to assess potential age-related differences in the outcomes. Additionally, the reliance on self-reported data during interviews for the diagnosis of hypertension and diabetes introduces the potential for recall bias. Finally, the recruitment of participants aged 45 and above may limit the broader applicability of the findings, necessitating further large-scale studies involving the general population.

## Conclusion

In this study, we found that increased baseline CVAI was positively associated with the risk of developing hypertension, and CVAI may applied as a useful tool for risk stratification of the hypertension incidence in middle-aged and older Chinese adults with a prediabetes stage.

## Data Availability

The raw data supporting the conclusions of this article will be made available by the authors, without undue reservation.
