# Corrigendum: Association between Chinese visceral adiposity index and risk of new-onset hypertension in middle-aged and older adults with prediabetes: evidence from a large national cohort study

**DOI:** 10.3389/fpubh.2025.1605866

**Published:** 2025-04-17

**Authors:** Lanlan Li, Linqiang Xi, Qianhui Wang

**Affiliations:** ^1^Department of Nephrology, Hospital of Xinjiang Production and Construction Corps, Urumqi, Xinjiang, China; ^2^Department of Nephrology, Second Affiliated Hospital, Medical School of Shihezi University, Urumqi, Xinjiang, China; ^3^Department of Cardiac Pacing and Electrophysiology, The First Affiliated Hospital of Xinjiang Medical University, Urumqi, China; ^4^Department of Cardiology, The Sixth Affiliated Hospital of Jinan University (Dongguan Eastern Central Hospital), Dong Guan, Guangdong, China

**Keywords:** prediabetes, hypertension, obesity, Chinese visceral adiposity index, biomarker

In the published article, there was an error regarding the affiliations for Linqiang Xi. As well as having affiliation 3, they should also have the affiliation “Department of Cardiology, The Sixth Affiliated Hospital of Jinan University (Dongguan Eastern Central Hospital), Dong Guan, Guangdong, China”. This has been added as affiliation 4.

The authors apologize for this error and state that this does not change the scientific conclusions of the article in any way. The original article has been updated.

